# Associations between eating habits and mental health among adolescents in five nordic countries: a cross-sectional survey

**DOI:** 10.1186/s12889-024-20084-w

**Published:** 2024-09-27

**Authors:** Anne-Siri Fismen, Leif Edvard Aarø, Einar Thorsteinsson, Kristiina Ojala, Oddrun Samdal, Arnfinn Helleve, Charli Eriksson

**Affiliations:** 1 Department of Health and Caring Science, Western Norway University of Applied Science, Bergen, Norway; 2 Department of Health Promotion, Norwegian Institute of Public Health, Bergen, Norway; 3 Department of Health Promotion and Development, University of Bergen, Bergen, Norway; 4 School of Psychology, University of New England, Armidale, NSW 2351 Australia; 5 Research Centre for Health Promotion, Faculty of Sport and Health Sciences, University of Jyväskylä, Jyväskylä, Finland; 6 Department of Learning, Informatics, Management, and Ethics, Karolinska Institute, Stockholm, Sweden

**Keywords:** Food habits, Meal habits, Adolescents, Mental health, Mental well-being

## Abstract

**Background:**

The role of eating habits in adolescent mental health has become a growing area of interest for researchers and health professionals. Recent studies suggest that healthy eating habits may play a role in the development and management of mental health. However, existing literature is largely based on clinical populations, and comparisons are challenged by sociocultural differences and methodological issues. The aim of the present study was to use nationally representative data based on standardized procedures, to explore associations between adolescents eating habits and mental health, considering the importance of age, gender, socioeconomic factors, and country.

**Methods:**

The study was based on data from Nordic adolescents (age 11, 13 and 15 years) who participated in the 2017/2018 Health Behavior in School-aged Children (HBSC) study (*n* = 22384). General linear modelling and multigroup regression analysis were used to examine the relationship between food habits (intake of fruit, vegetables, sweets, soft drinks), meal habits (intake of breakfast and having family meals together), and mental health (life satisfaction and subjective health complaints). The analyses were weighted and adjusted for age, gender, socio-economic status, and country.

**Results:**

Overall, healthier eating habits were associated with better mental health. The strongest associations were found between meal habits (breakfast consumption and having family meals together) and mental health. Gender and country differences were seen, with weaker associations found among Danish boys.

**Conclusion:**

Eating habits should be considered when promoting mental health in the adolescent population. While gender differences and cross-country variations exist, even minor enhancements in eating behaviors could yield meaningful benefits.

**Supplementary Information:**

The online version contains supplementary material available at 10.1186/s12889-024-20084-w.

## Introduction

 Good mental health and well-being during adolescence are critical to ensuring healthy transitions to adulthood, with implications for overall well-being, growth and development, and social and educational outcomes [1]. In the Nordic countries, the prevalence of child and adolescent mental illness appears comparatively lower than in numerous other Western countries [2, 3]. Nonetheless, recent trend studies indicate an increase in adolescents’ self-reported mental health complaints, and that the increase is more pronounced in the Nordic countries compared to other regions [4, 5]. Furthermore, trends of decrease in adolescent life satisfaction have been reported in most of the Nordic countries [6]. The etiology of these changes is not clear but do most likely involve a complex interplay of factors at both individual and contextual levels [7]. Furthermore, they are accompanied by trends of low and even decreased fruit and vegetable intake [8], increased intake of ultra-processed food [9], and, in Denmark and Sweden, also by less regular meal frequency [10]. Thus, the relationship between eating habits and adolescent mental health and well-being has become a growing area of interest for researchers and health professionals. Additionally, adolescents themselves strongly emphasize the link between eating habits and mental health [11].

Accumulating evidence from observational and intervention studies in nutritional psychiatry and psychology highlights the importance of diet for mental health development (9–12). Potential pathways underpinning these associations include those of the gut-brain axis and the role of specific nutrients in supporting brain function and neurotransmitter activity, which may have a positive effect on the development and management of mental health [12]. In this context, diet is the greatest modulator of the immune system-microbiota crosstalk, and much interest, and new challenges, are arising in precision nutrition as a way towards treatment and prevention [13]. However, while the impact of individual nutrients on mental illness is essential from a clinical perspective (9–12), the link between whole foods and general eating habits, and self-reported mental health and well-being, has a greater relevance in the field of public health. This broader perspective aims to more effectively address health benefits associated with eating habits and overall dietary intake in the general population. Importantly, eating habits may be largely modifiable, unlike other mental health risk factors (e.g., genetics), and are therefore highly relevant in promotion and prevention.

Increased intake of nutrient-dense foods such as fruits and vegetables has previously been linked to favorable impacts on mental health and well-being such as through higher levels of mental well-being [14]. However, the effect sizes were small and potentially mediated by socioeconomic status (SES) and other factors. A systematic review suggested that there might be an association between fruit and vegetable consumption and adolescents’ self-reported mental health and well-being [15]. Furthermore, consumption of sugar-sweetened beverages, fast food [16, 17], and other examples of ultra-processed food [18] are associated with symptoms of depression and anxiety, stress, and sleep dissatisfaction among children and adolescents. Beyond food habits, several studies link regular breakfast consumption and frequent family meals to adolescents’ mental health and well-being as well as to academic achievement [19–23]. Family routines like family meals are an essential part of family life and might offer consistency and a venue for checking in with family members. Family meals may thus act as contextual opportunities in which adolescents and their families can connect and strengthen their bonds [22]. As highlighted in the umbrella review by Snuggs et al. [24], sharing family meals may play a protective role in young people’s food habits and mental well-being, enhance the presence of family members, and foster a positive mealtime atmosphere. A meta-analysis suggested that skipping breakfast had somewhat an effect on increasing stress, psychological distress, and depression with mixed findings for anxiety [20].

Identifying determinants and pathways crucial for positive mental health among children and adolescents is underscored in the World Health Organization’s (WHO) mental health action plan [25]. However, the relationship between eating habits and mental health is complex, multifaceted, and not fully understood. Current evidence targeting both specific nutrients as well as whole foods and overall dietary patterns are largely based on clinical populations and comparisons are challenged by different conceptuality, sociocultural differences, and methodological issues. Studies based on nationally representative samples are scarce, and few studies allow for cross-country comparisons. Population-based studies are needed to explore this relationship and to inform public health interventions addressing adolescents’ mental health. Despite contextual similarities between the Nordic countries, cross-country differences are observed in e.g., adolescents’ food habits [26] and mental health [4, 5]. Cross-country comparisons can help identify the similarities and differences in associations between eating habits and mental health across different countries, which can provide insights into the underlying factors that contribute to these associations. Such research is the focus of an ongoing research collaboration on mental health among Nordic adolescents [27], represented by the authors behind this current study.

The Health Behavior in School-aged Children (HBSC) is a WHO collaborative cross-national study whose overall aim is to generate a greater understanding of health and health behavior and their context in the lives of young people [1]. These data have to a large extent been used to explore associations between mental health and a range of individual and contextual determinants [7]. However, as highlighted by Currie and Morgan [7], these data have not been used to investigate associations between mental health and eating habits in a cross-national sample. The aim of the present study was to use data from Nordic adolescents, aged 11, 13, and 15 years, participating in the 2018 HBSC study to explore associations between adolescents eating habits (food habits and meal habits) and mental health (health complaints and life satisfaction), considering the importance of age, gender, socioeconomic factors, and country.

## Methods

The study was based on nationally representative data from adolescents living in Denmark, Finland, Iceland, Norway, and Sweden, participating in the 2017/2018 HBSC study. The sampling procedure followed the HBSC standardized protocol (28) with the objective of achieving at least 1500 participants for each age group, assuming a 95% confidence interval of +/- 3% around a proportion of 50% and a design factor of 1.2, based on analyses of existing HBSC data [28]. The sampling unit of the participants varied slightly between the countries. In Finland, Norway, and Sweden, the primary sampling unit was the school class. In Finland, the probability proportional to size sampling method (PPS) was applied in school selection and the number of pupils in the schools was used as the measure of size. Within sampled schools, one class was randomly selected to participate. In Norway, the number of pupils at each class-level the previous school year was used to estimate the number of classes. In Sweden, a nationally representative sample of schools within the grades 5, 7, and 9 were randomly selected. Then a single class was randomly selected from each school according to instructions from Statistics Sweden. In Denmark, the primary sampling unit was the school. All students in the relevant grades were invited to participate. In Iceland, all schools in the country were invited to participate in the data collection to achieve the needed sample size. The students completed the HBSC internationally standardized questionnaire at school after receiving instructions from their teacher (28). The administration of the questionnaire used computers in Denmark, Finland, and Norway, paper-and-pencil in Sweden, and in Iceland both methods. Oral and written information outlining the confidentiality of their responses was provided, and participation was confidential and voluntary. Response rates varied across the countries. Schools/classes that declined to participate, and students who were absent on the day the survey was completed were the two main sources of nonresponse and were not followed up. The HBSC Data Management Centre checked the quality of the data collected, performed appropriate cleaning of the data, and merged national data sets into a Nordic data file. Detailed information about the study is available at www.hbsc.org.

### Measures

Self-reported health complaints and life satisfaction are understood as components of mental health [7]. These indicators are frequently used to measure adolescents’ self-reported mental health and well-being [1, 7]. In the present study, health complaints were measured by the HBSC Symptom Check List (HBSC-SCL); an eight item measure that may have both psychological and somatic origins [1]. Based on the HBSC Symptom Checklist (HBSC-SHC), the adolescents were asked how often they experienced the following symptoms over the past 6 months: headache, abdominal pain, backache, feeling low, irritability or in a bad mood, feeling nervous, sleeping difficulties, and dizziness. The five response categories were “About every day,” “More than once a week,” “About every week,” “About every month,” and “Rarely or never.” Consistent with standard procedures in HBSC (28), a recoded mean score was constructed (sum score divided by the number of items with valid data) and ranged from 1(Rarely or never any symptoms) to 5 (All eight symptoms about every day). The HBSC-SHC has adequate test–retest reliability and validity properties [29]. Cronbach’s alpha for the HBSC-SHC was 0.84.

Life satisfaction is defined as “A cognitive global judgment of one’s life as a whole” [30]. Participants rated their life satisfaction using a single-item question referred to as the Cantril ladder [31]: “Here is a picture of a ladder. The top of the ladder ‘10’ is the best possible life for you and the bottom ‘0’ is the worst possible life for you. In general, where on the ladder do you feel you stand at the moment?” The Cantril ladder has shown good reliability and convergent validity among adolescents [32].

Eating habits was measured by food habits (fruit, vegetables, sweets, and sugar-sweetened soft drinks consumption) and meal habits (breakfast consumption and having family meals together). Food habits was measured by questions on frequency of intake: “How many times a week do you consume fruit/vegetables/sweets/sugar-sweetened soft drinks?” The response categories were ‘Never’, ‘Less than once a week, ‘Once a week’, ‘Two to four times a week’, ‘Five to six times a week’, ‘Once a day’, and ‘More than once a day’. For the present study, the response categories were recoded into ‘Never’ = 0; ‘Less than once a week’ = 0.5; ‘Once a week’ = 1.0; ‘Two to four times a week’ = 3.0; ‘Five to six times a week’ = 5.5; ‘Once a day’ = 7.0; ‘More than once a day’ = 10.0). The question, which is part of the HBSC food checklist, is a recognized as valid instrument in epidemiological studies ranking adolescents according to their usual food intake [33]. Meal habits was measured by questions on breakfast consumption and family meals. Breakfast consumption: “How often do you usually have breakfast (more than a glass of milk or fruit juice)? Please tick one box for weekdays and one box for weekends” The response categories were ‘I never have breakfast during the week’ (0), ‘One day’ (1), ‘Two days’ (2), ‘Three days’(3), ‘Four days’ (4) and ‘Five days’ (5) for schooldays; and ‘I never have breakfast during the weekend’ (0), ‘I usually have breakfast on only one day of the weekend (Saturday OR Sunday)’(1) and ‘I usually have breakfast on both weekend days (Saturday AND Sunday)’ (2). The responses were summed to a total range of days of eating breakfast (0–7 days a week). Family meals: “How often do you and your family usually have meals together?” The response categories were ‘Every day’, ‘Most days’, ‘About once a week’, ‘Less often’, ‘Never’. The responses were recoded and summed to a total range of family meals over a week: ‘Every day’ =7, ‘Most days’ = 5, ‘About once a week’ = 1, ‘Less often’ = 0.5’, ‘Never’ = 0.5. The item is considered a reliable measure to address family meals in a Nordic context [34].

Socioeconomic status was measured by the family affluence scale which comprises six items and is a measure of material affluence derived from the characteristics of the family’s household. In line with the HBSC recommendations [35], SES was categorized into the lowest (20%) middle (60%), and highest (20%) groups, based on the country’s FAS score distribution.

### Statistical analyses

Data were weighted to compensate for over-sampling of Swedish-speaking adolescents in Finland. Data were also weighted to ensure equal representation across gender, age, and countries while preserving the total number of observations. After weighting of data, the number of observations in each subgroup defined by gender, age, and country was 738. The total number of observations before weighting was 22,384 (247 with information missing on gender). After weighting the total number of observations was 22,385, or 22,140 with those with missing on gender excluded. In all statistical analyses, adjustments were made for cluster effects. In Iceland, the school authorities in Reykjavik did not approve registering of schools or school classes. Therefore, all schools in Reykjavik were treated as belonging to one cluster (per age).

Preliminary analysis (shown in Supplementary Table 1) indicated that some eating habits variables were moderately correlated; breakfast during weekdays and breakfast during weekends (*r* = .368), (fruit consumption and vegetable consumption (*r* = .550), and sweets consumption and soft drink consumption (*r* = .523). All other correlations were small, in the range of 0.040 to 0.151. Three summary scores, one for each pair of variables were therefore constructed and used in the multigroup analyses comparing mean scores across subgroups.

To produce summary variables covering all eating variables combined, global indices for healthy eating, general linear modelling (GLM) was run with all seven eating variables as predictors and the health complaints sum-score and life satisfaction as outcomes. The predicted values were saved and recoded into deciles. A distinction could be made between meal habits (breakfast weekdays, breakfast weekends, family meals) and food habits (consumption of fruit, vegetables, sweets, soft drinks), and summary variables were constructed for (i) meal habits predicting health complaints, (ii) food habits predicting health complaints, (iii) meal habits predicting life satisfaction, and (iv) food habits predicting life satisfaction. These variables were not recoded into deciles but standardized and used in a series of regression models conducted with Mplus.

The score for (i) meal habits (predicting health complaints) and the score for food habits (ii) (predicting health complaints) were used in a multigroup regression model with health complaints as the dependent variable and with adjustment for age. Four groups were defined (a) boys from all countries except Denmark, (b) boys from Denmark, (c) girls from all countries except Denmark, and (d) girls from Denmark. The decision to distinguish between Danish boys and girls, and boys and girls from the other Nordic countries, was made after a series of GLM analyses in SPSS Complex which confirmed that such a distinction was required. Similar analyses were performed in multigroup regression model including iii) meal habits (predicting life satisfaction), and iv) food habits (predicting life satisfaction).

Most of the statistical analyses were carried out with the GLM procedure in SPSS Complex. Complex allows for simultaneous weighting of data and adjustments for cluster effects. Some regression models as well as all multigroup testing of these models were done in Mplus with the MLR estimator, with weighting and adjustments for cluster effects.

## Results

Table 1 shows mean score (M) and standard deviation (SD) on mental health and eating habits by gender. Supplementary Table 1 shows correlations between the items.

**Table 1:** Mental health and eating habits by gender

	Boys		Girls		*n*
	*M*	*SD*	*M*	*SD*	unweighted
Mental health					
Health complaints (1–5)	1.95	0.75	2.32	0.88	21 713
Life satisfaction (0–10)	7.88	1.75	7.44	1.92	21 780
Food habits					
Fruit consumption (0–10)	4.43	3.15	5.05	3.11	21 794
Vegetable consumption (0–10)	4.87	3.09	5.54	3.02	21 689
Fruit and vegetable consumption combined (0–10)	4.65	2.75	5.30	2.69	21 809
Sweets consumption (0–10)	2.20	2.03	2.25	1.92	21 684
Soft drinks consumption (0–10)	2.22	2.31	1.57	1.93	21 694
Sweets and soft drinks consumption combined (0–10)	2.21	1.92	1.91	1.66	21 705
Meal habits					
Breakfast weekdays (0–5)	4.06	1.68	3.86	1.80	21 873
Breakfast weekends (0–2)	1.72	0.60	1.74	0.57	21 728
Breakfast weekdays and weekends combined (0–7)	5.78	1.97	5.60	2.07	21 624
Family meals (0–7)	5.12	2.11	4.88	2.12	21 750

The GLM analysis indicated that mean scores on mental health and eating habits were associated with gender, age, SES, and country (Table 2). Girls reported lower life satisfaction and more health complaints, higher fruit and vegetable consumption, lower soft drink consumption, less frequent breakfast during weekdays, and less frequent family meals than did boys. Older, compared to younger, adolescents reported lower life satisfaction and more health complaints, lower fruit consumption, higher sweets and soft drink consumption, less frequent breakfast consumption, and less frequent family meals. Vegetable consumption was less strongly and less consistently associated with age. Higher SES was associated with lower levels of health complaints and higher life satisfaction, as well as higher fruit and vegetable consumption, lower soft drink consumption, and more frequent meals. Higher levels of health complaints, as well as lower scores on life satisfaction, were seen in the Swedish sample. More frequent vegetable consumption, breakfast consumption, and family meals were seen in the Danish sample, while higher fruit consumption as well as lower sweets and soft drink consumption were seen in the Icelandic sample.

**Table 2:** Mean score on mental health and eating habits by gender, grade, SES, and country

	Health complaints		Life satisfaction		Fruit consumption		Vegetable consumption		Sweets consumption		Soft drink consumption		Breakfast weekdays		Breakfast weekends		Family meals	
Measure	*M*	*p*	*M*	*p*	*M*	*p*	*M*	*p*	*M*	*p*	*M*	*p*	*M*	*p*	*M*	*p*	*M*	*p*
Gender																		
Boys	1.96	< 0.001	7.83	< 0.001	4.47	< 0.001	4.83	< 0.001	2.20	0.097	2.25	< 0.001	3.98	< 0.001	1.71	0.020	5.07	< 0.001
Girls	2.32		7.40		5.10		5.50		2.25		1.60		3.78		1.73		4.83	
Age																		
11 years	2.00	< 0.001	7.99	< 0.001	5.15	< 0.001	5.26	0.015	1.96	< 0.001	1.68	< 0.001	4.19	< 0.001	1.79	< 0.001	5.25	< 0.001
13 years	2.15		7.53		4.67		5.05		2.30		1.96		3.88		1.70		5.01	
15 years	2.28		7.32		4.54		5.19		2.43		2.13		3.57		1.65		4.59	
SES																		
Low	2.19	< 0.001	7.37	< 0.001	4.40	< 0.001	4.70	< 0.001	2.25	0.745	2.04	< 0.001	3.68	< 0.001	1.67	< 0.001	4.76	< 0.001
Middle	2.12		7.68		4.70		5.21		2.22		1.87		4.02		1.74		5.07	
High	2.11		7.96		5.23		5.78		2.20		1.80		4.13		1.77		5.09	
Country																		
Denmark	1.99	< 0.001	7.60	< 0.001	5.14	< 0.001	5.75	< 0.001	2.45	< 0.001	2.09	< 0.001	3.89	< 0.001	1.79	< 0.001	5.36	< 0.001
Finland	2.22		7.79		4.25		4.67		2.08		1.88		3.87		1.71		4.06	
Iceland	2.20		7.54		5.36		4.70		1.85		1.42		3.78		1.59		5.32	
Norway	1.99		7.71		4.86		5.10		2.31		2.20		3.92		1.77		5.10	
Sweden	2.30		7.44		4.33		5.61		2.45		2.03		3.93		1.73		4.90	

*p*-values adjusted for cluster effect

As shown in Table 3, higher fruit consumption, lower sweets consumption, as well as more frequent breakfast consumption (weekdays and weekends), and family meals were associated with lower levels of health complaints and higher life satisfaction. Lower soft drink consumption was associated with lower levels of health complaints. No significant associations were found between soft drink consumption and life satisfaction, nor between vegetable consumption and any of the two mental health measures. The associations were small, with the largest between breakfast consumption on weekdays and health complaints (*r* = − .124, *p* < .001) and family meals and life satisfaction (*r* = − .121, *p* < .001).

**Table 3:** Health complaints and life satisfaction by eating habits, adjusted for gender, age, country, SES

Eating habits times per week	Health complaints^1^		Life satisfaction^2^	
	*r*	*p*	*r*	*p*
Fruit consumption	− 0.044	< 0.001	0.108	< 0.001
Vegetable consumption	0.000	0.997	0.016	0.084
Sweets consumption	0.073	< 0.001	− 0.035	0.001
Soft drink consumption	0.072	< 0.001	− 0.016	0.072
Breakfast weekdays	− 0.124	< 0.001	0.106	< 0.001
Breakfast weekends	− 0.102	< 0.001	0.109	< 0.001
Family meals	− 0.100	< 0.001	0.121	< 0.001

^1^*R*^2^ = 0.07

^2^*R*^2^ = 0.131

**Fig. 1:**
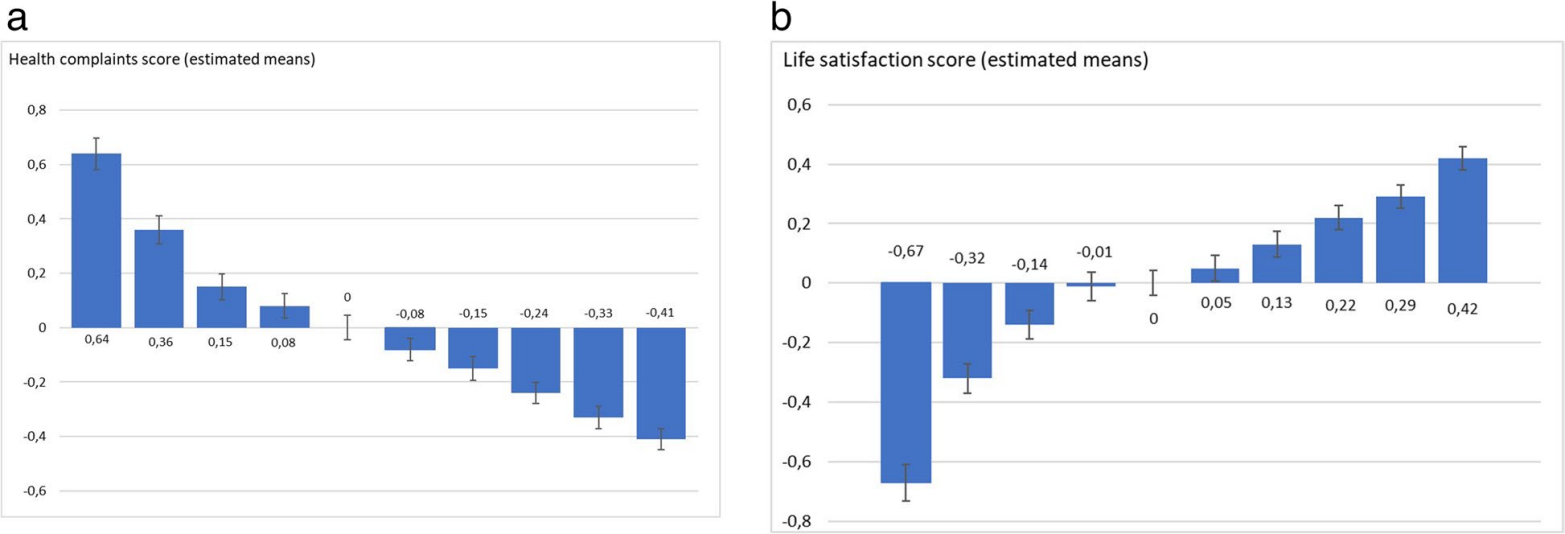
**a** and **b**. Health complaints score and life satisfaction score by healthy eating (predicted values in deciles)

**Fig. 2:**
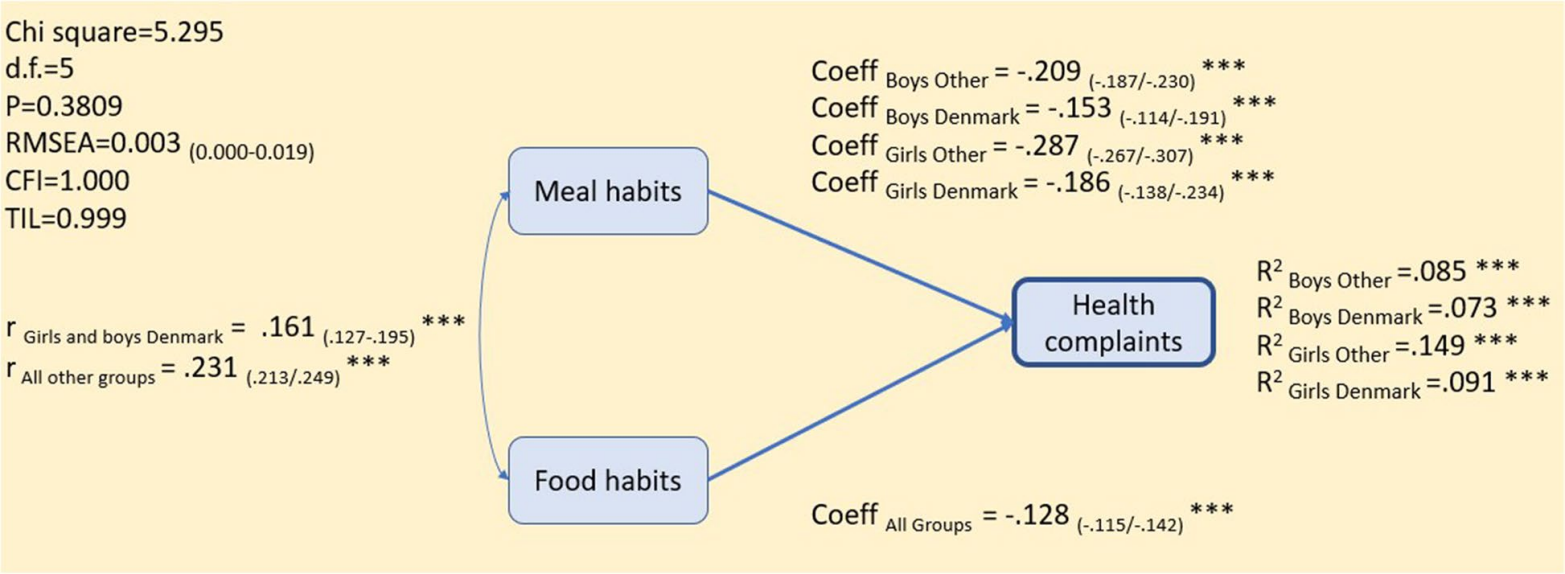
Health complaints by eating habits (*N*_FULL MODEL_ = 22,384) adjusted for age and SES

Figure 1a and b illustrates a close to linear relationship between healthy eating habits (predicted values recoded into decentiles) and health complaints and life satisfaction (standardized), and show that the healthier eating habits, the lower score on health complaints and higher life satisfaction. The association was close to linear across the last seven categories, but slightly steeper over the first three (less healthy eating habits). The difference between the top and bottom scores were 1.05 (health complaints) and 1.09 (life satisfaction). Given that the dependent variable was standardized, this value can be interpreted as a z-value. This means that the difference between the two extreme categories is large. When adjusting for demographic predictors (see Fig. 2), the difference between the extreme groups on the eating habits scale was marginally smaller for health complaints (0.95) and life satisfaction (1.05).

As shown in Fig. 2, the multigroup regression model indicated that healthy food habits were negatively associated with health complaints, and there were no significant differences in the strength of this association across subgroups defined by gender and country (Denmark versus all the other Nordic countries) (*r* = − .128). Meal habits, compared to food habits, was generally more strongly associated with health complaints, but there was considerable variation across subgroups. Absolute association values were stronger among girls than among boys, and less strong in Denmark compared to the other countries.

**Fig. 3:**
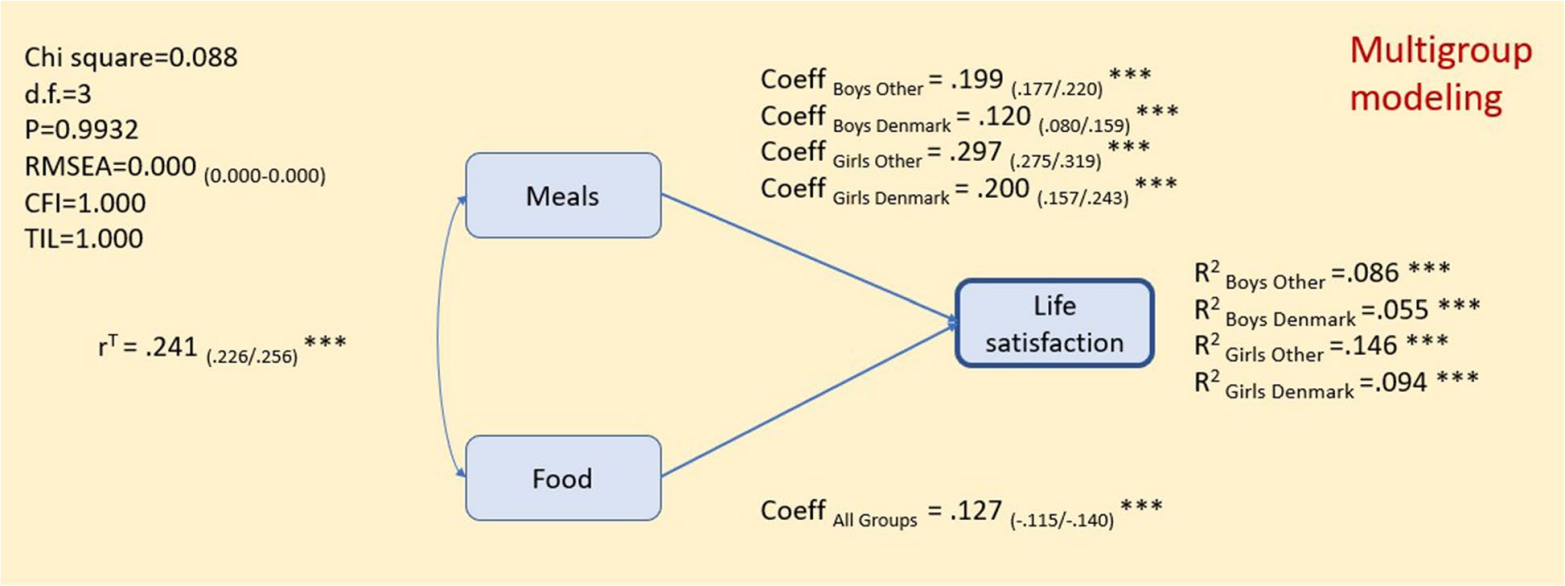
Life satisfaction by eating habits (*N*_FULL MODEL_ = 22,384) adjusted for age and SES

As shown in Fig. 3, healthy food habits were positively associated with life satisfaction and there were no noticeable differences across countries (*r* = .121). Healthy meal habits, compared to food habits, was more strongly associated with life satisfaction except for Danish boys, but there was considerable variation across subgroups. Absolute associations were higher among girls than among boys and lower in Denmark compared to the other countries.

## Discussion

The present study suggests that there may be limited associations between eating habits and mental health in Nordic adolescents, with gender differences and cross-country variations observed in the associations between meal habits and health complaints and life satisfaction.

The association between fruit and vegetable consumption and mental health may be elucidated in the context of recent studies, which indicate that a higher intake of these foods may be associated with increased life satisfaction [14], reduced psychological distress [15] and other favorable outcomes of mental health in children and adolescents [15, 36] Furthermore, there was a weak inverse relationship between reduced sugar consumption and improved mental health in the present study, supporting results from previous studies in which it was concluded that high-calorie, low-nutrient value unhealthy foods are associated with increased odds of psychological distress in children and adolescents [37] and higher levels of stress and depressive symptoms [17]. In contrast, a systematic review suggested that chocolate consumption may lead to lower levels of depressive and anxiety symptoms in the short term [38]. This may be attributed to chocolate’s content of (poly)phenolic compounds and its positive impact on mood, depression, and other mental health outcomes [39].

The present study suggests that having family meals together may be associated with reduced health complaints and increased life satisfaction, which is in line with previous studies [20, 24]. Furthermore, also in line with previous research, girls, older adolescents, and adolescents living in families with lower SES, reported higher levels of health complaints and lower life satisfaction [40], while boys, younger adolescents, and adolescents living in families with higher SES reported more favorable food habits [1].

### The role of eating habits in adolescent mental health

The findings of healthy eating habits as a predictor of better mental health may be explained by the hypothesis that food items high in essential nutrients are crucial for proper brain functioning and development [12]. This importance of essential nutrients extends to also explain the observed link between meal habits and mental health. Breakfast consumption and family meals are proposed to offer nutritional advantages, as regular breakfast consumption and sharing meals are associated with favorable dietary patterns in children and adolescents, including the consumption of fruits, vegetables, and various nutrients [19, 41, 42]. This perspective may explain the observed correlations between meal habits and food habits. However, while the present study indicates a consistent association between food habits and mental health across genders and countries, both gender and country differences emerged in the relationship between meal habits and mental health. This may indicate that food habits and meal habits involve different mechanisms. Whereas the consistent relationship between food habits and mental health leans towards an explication through essential nutrients and biochemical mechanisms [12], the importance of meal habits is more likely to be explained by sociocultural processes. Family meals can provide a context for parental support, parental modeling, and parental communication [24], serving as a platform to address various aspects of adolescents’ lives and develop coping strategies for daily challenges. Parental communication is previously suggested to positively impact children’s health [43] and to mediate the relationship between family meals and mental health [44]. This may explain the observed association between meal habits and health complaints and life satisfaction. However, it should be noted that family structure may act as a confounder in this relationship, as adolescents living in single-parent households may have less favorable food habits [45] and poorer mental health [46].

The observed associations between eating habits and mental health were statistically significant but small. Nevertheless, a close-to-linear pattern across categories was demonstrated which aligns with previous research [14, 44, 47]. Increasing fruit and vegetable consumption, as well as the frequency of breakfasts and family meals, may have positive effects on adolescent mental health and well-being, and even minor improvements in eating habits may have population-level benefits, suggesting the need for prioritizing interventions and promoting frequent family meals. Green and yellow vegetables, along with fresh fruit, should be emphasized as they may be particularly beneficial to adolescents’ general mental health [15], as well as contributing to the prevention of several chronic diseases [48, 49]. However, it is essential to note that a recent systematic review and meta-analysis concluded that young people (below 26 years) following vegan or vegetarian diets are at higher risk of depression and anxiety [50]. Another systematic review [51] concluded that the evidence on the effect of vegetarian and vegan diets on depression is contradictory, possibly due to the heterogeneity of the studies analyzed. This underlines the importance of exploring the nuances of diets and food habits in greater detail, particularly in the relationship with mental health, encompassing dietary composition, cultural beliefs, and economic conditions.

### Gender differences and cross-country variation

In the present study, the strength of the association between family meals and mental health was stronger among girls than among boys. This finding aligns with the findings of a comprehensive systematic review addressing these effects [19]. The specific mechanisms explaining gender differences are not fully understood, but girls may exhibit distinct responses to family dynamics, particularly concerning the frequency of family meals and its impact on mental health. If the positive effects of family meals are attributed to factors such as family connectedness and communication, it is plausible that boys may not experience comparable protective benefits from frequent family meals, owing to a different and possibly lower responsiveness to familial dynamics compared to girls [52]. However, it is crucial to note that family meals are not necessarily synonymous with enjoyable dining experiences with company and in supportive environments. Moreover, scientific support exists also for alternative pathways, namely that mental health can influence eating habits. Negative emotions like sadness and stress have been linked to unhealthy food habits [38, 53–55], and it is plausible that adolescents with better mental health are more likely to engage in family meals. Furthermore, a simulation-based study revealed that reinforcing loops involving perceived pressure on psychosocial stress aggravated the negative energy-balance related behaviours such as emotional eating [56]. This underlines a possible bidirectionality relationship between eating habits and mental health.

The family environment was consistently related to mental health (life satisfaction) in a previous study among adolescents in 45 countries [57]. In the present study of five Nordic countries, the associations between meal habits and mental health were significant in all countries. However, the association between meal habits and mental health was weaker among Danish boys and girls compared to their counterparts living in other countries. The observed variability may be viewed in light of disparities in breakfast quality and cultural distinctions across the Nordic study populations [58]. Furthermore, while Danish adolescents report approximately the same levels of perceived social support from parents as do adolescents from the other Nordic countries, they report the highest levels of perceived social support from peers [1]. It is suggested that in Denmark, compared to other Nordic countries, people spend more time together with friends (https://www.statista.com/statistics/522039/time-spent-visiting-friends-countries/). This may indicate that among Danish adolescents, social contexts and relations outside the family may compensate for the influence of parents and siblings. However, more research is needed to explore this.

### Strengths and limitations

The strengths of the present study are the large number of participants, the use of nationally representative data and standardized methods of the survey allowing cross-country analyses. Furthermore, all data were weighted to ensure equal representativeness across age, gender, and country. The study adds to the HBSC review of determinants of mental health [7], in which the need for the HBSC study to explore the association between eating habits and mental health was highlighted. Furthermore, this is the first study using HBSC data to provide a cross-country comparison perspective, and the use of advanced statistical methods enabled analyses of differences between the five Nordic countries. Another strength is the inclusion of both food habits and meal habits, as previous studies typically focused on one or the other. Due to the cross-sectional nature of the data, establishing causation is unattainable. There is a need for more longitudinal studies that track changes in eating habits over time to predict mental health outcomes. These studies need to focus further on SES and parent-adolescent communication factors.

## Conclusion

This study emphasizes the multifaceted nature of the association between eating habits and mental health in Nordic adolescents. Gender differences and cross-country variations further highlight the need for a nuanced approach in understanding and addressing the mechanisms in the relationship between eating habits and mental health. In conclusion, the study provides valuable insights that can inform clinicians as well as public health policies and interventions addressing mental health in the adolescent population.

## Supplementary Information

Supplementary Material 1.

## Data Availability

The datasets used and/or analyzed during the current study are available from the corresponding author on reasonable request. Please contact dmc@hbsc.com.
