# Supplementary material for: Associations between eating habits and mental health among adolescents in five nordic countries: a cross-sectional survey
Source: BMC Public Health. 2024 Sep 27;24:2640. doi: 10.1186/s12889-024-20084-w (PMC11438251; doi:10.1186/s12889-024-20084-w)
Supplement: Supplementary file 1 — Supplementary Material 1. [file 12889_2024_20084_MOESM1_ESM.docx]

**Supplement Table 1** Correlations ^1,2,^ between eating variables (n _WEIGHTED_ = 21,630-22,072).

| Measure | 1 | 2 | 3 | 4 | 5 | 6 |
| --- | --- | --- | --- | --- | --- | --- |
| 1. Breakfast during weekdays |  |  |  |  |  |  |
| 2. Breakfast during weekends | .368 |  |  |  |  |  |
| 3. Fruit consumption | .136 | .137 |  |  |  |  |
| 4. Vegetable consumption | .145 | .151 | .550 |  |  |  |
| 5. Sweets consumption | -.097 | -.066 | -.040 | -.044 |  |  |
| 6. Soft drink consumption | -.143 | -.113 | -.120 | -.124 | .523 |  |
| 7. Family meals | .145 | .128 | .110 | .130 | -.064 | -.061 |

^1^ All correlations were significant (*p* < .001). Significance testing with weighting and adjustment for cluster effects.

^2^ The correlation between ‘Health complaints’ and ‘Life satisfaction’ (weighted) was -.481 (*p* < .00
